# Integrating strategic and tactical decisions in livestock supply chain using bi-level programming, case study: Iran poultry supply chain

**DOI:** 10.1371/journal.pone.0185743

**Published:** 2017-10-05

**Authors:** Ebrahim Teimoury, Armin Jabbarzadeh, Mohammadhosein Babaei

**Affiliations:** Department of Industrial Engineering, University of Science and Technology, Tehran, Iran; Southwest University, CHINA

## Abstract

Inventory management has frequently been targeted by researchers as one of the most pivotal problems in supply chain management. With the expansion of research studies on inventory management in supply chains, perishable inventory has been introduced and its fundamental differences from non-perishable inventory have been emphasized. This article presents livestock as a type of inventory that has been less studied in the literature. Differences between different inventory types, affect various levels of strategic, tactical and operational decision-making. In most articles, different levels of decision-making are discussed independently and sequentially. In this paper, not only is the livestock inventory introduced, but also a model has been developed to integrate decisions across different levels of decision-making using bi-level programming. Computational results indicate that the proposed bi-level approach is more efficient than the sequential decision-making approach.

## Introduction

With the introduction of the concept of supply chain management in the early 1980s, inventory management was also recognized as one of the most important drivers of supply chain. At first, most inventory studies in the field of inventory management focused exclusively on non-perishable inventories, but soon after, the concept of perishable inventory was proposed. Recognizing the importance of perishable inventory in human lives, many researchers have studied mathematical models to optimize perishable products policies in the supply chain [1–4].

One of the most important types of perishable inventories are protein products. With careful examination of these type of products, it is clear that livestock inventory constitutes an important component in the supply chain of this products. For example, consider the red meat supply chain. This supply chain is composed of two parts: the livestock supply chain and the cold supply chain. The livestock supply chain includes the processes and steps associated with nurturing the animal from birth until slaughter; while the cold supply chain refers to the processes and steps that take place after slaughter. So far, researchers have focused almost exclusively on the cold part of supply chain [5–10]. Though the cold supply chain processes and steps are important, more than 80% of the time needed from the first step of supply chain until delivery of the final product to the customer occurs when the inventory is still alive [11]. Nevertheless, most researchers focus on cold part of supply chain and just consider perishable products and few researches focus on livestock inventory in supply chain. In most of this few researches, conceptual models are presented by researchers in the fields of agriculture and animal husbandry and little attention has been paid to optimization models and economy aspect of problem. Therefor, this paper presents a new optimization model with consideration of parameters and decision variables that are specific for livestock supply chain. Specifically, this article considers the chicken supply chain, which is one of the most prominent livestock supply chains. On the other hand, decisions can be classified into three categories: strategic, tactical and operational [12]. Some models are developed to make dicision in just one category and some models are developed to make decision in more than one category. When there is a need to make decision in different category simultiniusely, for example deciding about location (strategic one) and price (tactical one), Most articles use sequential approach. In other words, first strategic decisions are made, and then, using the outputs of these decision variables, tactical and operational decisions are made sequentially. However, in this approach, interactions between dicisions are ignored even when there is a relationship among the variables of different categories. Thus, in this paper, not only is the livestock inventory introduced, but also a model has been developed to integrate decisions across different levels of decision-making using bi-level programming.

It should be noted that multi-level models are a special type of stakleberg game and are often used to model competition situations in which one of the players has more power and can make the first move. Because strategic decisions have more impact on cost and time than the other decision-making categories (i.e., tactical and operational decisions), strategic decisions can be considered as the leader while tactical and operational decisions constitute the followers. Different approaches have been used to solve multi-level models. In this article, fuzzy goal programming presented in [13] is used to solve the model. Comparing the results of this decision-making approach with a common sequential approach, which involves determining policies using different decision variables in tandem, indicates the efficiency of the proposed approach.

In section 2 of this article, the poultry supply chain, as one of the most important livestock supply chains, is investigated, and differences between livestock and non-livestock supply chains are studied. Next, the important but understudied variables that exist in such supply chains are examined. Section 3 presents a literature review and analyzes the gap in this research area to be filled by the present paper. Problem is described in section 4. A mathematical model to integrate the strategic and tactical decisions is proposed in section 5. Solution method and computational results are presented in sections 6 and 7 and finally, the conclusion is presented in section 8.

### Livestock supply chain

**Fig 1:**
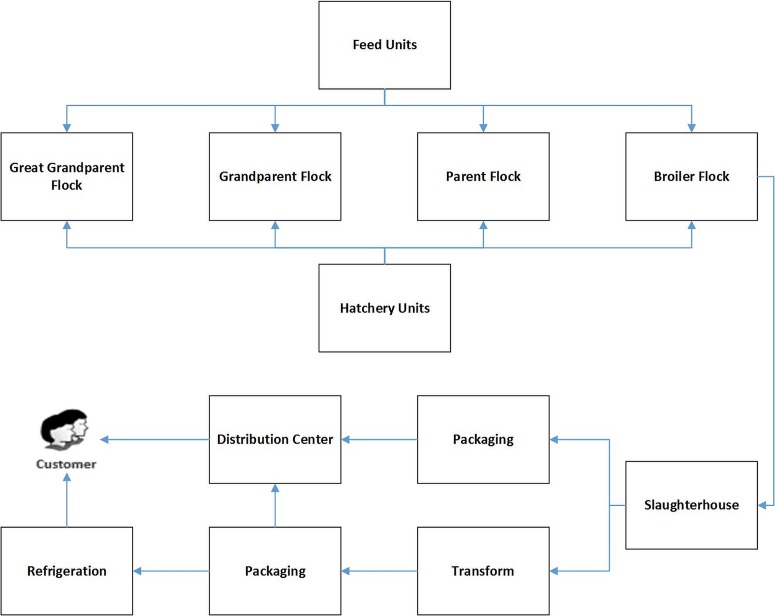
Meat poultry supply chain.

The supply chain of protein products is amongst the most important and influential supply chains human beings must deal with. In some protein products’ supply chains including red meat, fish meat, and chicken meat, the inventory flow is divided into two parts: livestock inventory flow and cold inventory flow. All the steps from birth, and even before birth, involved in delivering protein goods such as packaged meat, eggs, and sausages, are part of this supply chain. To distinguish between the livestock parts and the cold parts of the supply chain, the meat chicken supply chain is considered here. As shown in Fig 1, this chain is composed of different units both in the livestock and cold sections.

The first step in the process of producing chickens is called pure line. Line chickens are the only chickens in the process that can produce their own counterpart. In the next level, grandparent chickens can be seen. The product of grandparent chickens is called a parent. After that, broiler flocks are produced. These four stages are the main stages of the meat chicken livestock supply chain. It should be noted that food supply units and hatchery units also have an important role in this supply chain. Food supply units, supply the food for diferent poultry farms and hatchery units can raise the different type of chickens like grandparent, parent of broiler chickens. After broiler, the chickens are sent for slaughter. The slaughter of living creatures, such as chickens, shifts the nature of the chain from a livestock chain to a cold chain. As illustrated in Fig 1, after the slaughtering of the poultries, based on the product, different stages are performed to deliver the products to the end customer.

Special conditions and specific decision variables that exist in the livestock supply chain generate substantial differences between the livestock and the cold parts of the supply chain. One of the most important differences is growth and its side effects. The growth curve of the chicken indicates an obvious difference in growth rate over time. If the chickens are slaughtered they are younger, the number of hatching and breeding periods will be more. On the other hand, by maintaining each chicken for a longer period, benefits from scaling and prorating the fixed costs of breeding like less day-old chicken buying cost can be obtained. Thus, answering the question of the optimal age for slaughter considering different conditions is one of the central issues studied in this article. Another difference between livestock inventory and other types of inventory like non-perishabe inventory, is the mortality rate. Different breeding conditions lead to different rates of mortality in the animals. Planning must be conducted based on this rate. Moreover, the performance of these supply chains can change over time. For example, laying hens encounter reductions in their egg-laying rate as they age. On the other side, aging and the increased body weight cause a change in the costs imposed by time. Because more body weight cause in more feed consumption rate.

Mentioned differences between livestock and non-livestock inventories necessitate independent attention to livestock supply chains. Thus, the literature available in the field of livestock inventory supply chains is reviewed and the use of multi-level models in decision-making process is studied in the following section.

### Literature review

Little research has been conducted on livestock supply chain management for optimization, and most research studies in this area are limited to qualitative issues. To increase profit by identifying potential areas for improvement, Shamsuddoha [14] studied a chicken supply chain in both forward and reverse supply chains using system dynamics methodology. Heft-Neal et al. [15] examined three different structures of the chicken supply chain in Thailand, which include informal and rural structures, industrial structures, and formal structures. Studies conducted on these three structures produced reliable data, which was used to identify the advantages and disadvantages of each system and select the optimum structure. Iifft et al. [16] studied different structures of chicken supply chains in Vietnam, producing a simple mathematical model to determine the optimum production amount in the different structures. Mohammed et al. [17] conducted a study entitled, “Measuring Competition in Chicken Supply Chain in Malaysia”, in which the researchers evaluated competitiveness indices in supply chains using a structural assessment model. The used indices are more manufacturing related, and the main assessment is based on the production amount in Malaysia. He also compared integrated and non-integrated supply chains in terms of production amount and meeting customers’ demands.

Darivandi et al. [18] has discussed designing a meta system for the meat chicken supply chain structure in Iran. This has been done using functionalist, interpretive, and liberating paradigms. Besides, VSM and SCOR tools have been used to analyze the chain’s situation. Finally, a novel model was developed for the supply chain management body based on Ikaf’s organization theory. Various aspects of chicken supply chain risks in India have been evaluated by Mohan et al. [19]. Risks of raw materials were recognized as the most important risks in this supply chain. Then, various stakeholders of supply chains were identified, and their possible impacts and reactions were examined. The conclusion of this study also emphasized the necessity of chicken supply chain data integration for rapid and efficient response to disasters such as avian flu. Shamsuddoha et al. [20] presented a conceptual model of reverse logistics to reduce the environmental impact and increase sustainability in the chicken supply chain in Bangladesh.

Sana [21] developed a mathematical model to determine the fish and ducks’ optimal price in a common supply chain. In the target supply chain, fish supply chain outputs in duck supply chain and vice versa can be used as food. It is assumed that demand is a function of price and on-hand inventory. Given that agricultural and livestock products harm the land through greenhouse gas emissions and biological changes, Stehfest et al. [22] attempted to measure the impact of different policies on the increase or decrease of the agricultural products’ environmental impact of the production supply chain using two economic models, Impact and Lito, and create an integrated model. Rebolledo et al. [23] also investigated the destructive environmental impacts of living inventory supply chains in Vietnam. The authors attempted to represent a developed methodology to quantify and measure the amount of ammonia released for raising a living creature and presented a strategy to select a breeding ground so that emissions are reduced. A capacitated vehicle routing model to reduce the emission of greenhouse gas is presented by Kim et al. [24]. The results of total routing distance is compared in three scenarios; current situation, capacitated vehicle routing and dynamic capacitated vehicle routing.

A goal programming model is developed by Sauian and Othman [25] to optimize the product combination of livestock supply chains in cases of seasonal and non-seasonal situations. Napel et al. [26] introduced strategies to increase the stability value in poultry supply chains by developing a system of animal production and husbandry. Stable strategies refer to methods that increase the sustainability of the production system and maximize its stability.

Lapar et al. [27] developed a Bayesian approach for retailers to determine whether to enter the market. Retailers can make this decision in accordance with available resources and establish their supply chain quality level for the market. Supply chain quality determines supply type or the living inventory besides determining quantity. Barge et al. [28] developed some strategies to determine the best practice to apply RFID divices in livestock management. Then, with regards to the type and structure of the supply chain in terms of number and size, they evaluated the operating system in the meat supply chain.

Table 1, categorizes the researches in the field of livestock inventory management. According to this table there is different approaches, some researchers consider one facility and some of them consider two or more as supply chain. Then the problem modeling approaches are studied. The type of problem, specifies another classification. The purpose of some researches is to make the appropriate decisions, and others to analyze the current situation. Finally, it is pointed out that articles whose purpose are making appropriate decisions, whether considered the interaction of different decisions or not.

**Table 1:** Classification of the literature dedicated to livestork inventory modelling contex.

Author/s	Approach		Modelling Approach				Type					Interaction between Members	
							Decision				Analysis		
							Decision Type/s			Decision Variable			
	SCM	Single Facility	Mathematical Modeling	Conceptual Modelling	MCDM	Other	Strategic	Tactical	Operation			Yes	No
[14]	●					System Dynamis	●						●
[15]				●							●		●
[16]	●		●					●		Production Volume			●
[17]	●					Structual EquationModeling					●		●
[18]	●					System Dynamics	●						●
[19]	●				●						●		●
[20]	●				●		●						●
[21]	●		●					●		Price			●
[22]	●				●						●		●
[23]		●					●				●		●
[24]	●		●				●			Routing			●
[25]		●	●				●			Product Mix			●
[27]	●					Bayesian Approach	●			Market Selection			●

According to this table, it is clear that there are very few number of articles that attempt to optimize the variables which are specially for livestock inventories like breeding period. Among these papers, decision variables such as price and production amount, are considered that not only they are not exclusive for the livestock inventory but also, they are considered in optimizing other types of inventories like perishable one. On one hand, it is clear that in none of the reviewed articles, the interaction of different decisions with which supply chain managers confront, is not addressed. On the other hand, it is even more important that even the articles which develop optimization models for livestock inventory, are not considered some of the major differences between livestock inventory and other types of inventory. For example, weight changes over time or mortality rates is important points that are not considered in these papers. Therefore, in this paper, a bi-level model is presented. In this model livestock supply chain issues and exclusive parameters are considered. In addition, the real situation’s decision-making variables that are less considered in the articles, such as the optimal breeding period, are came into account. Although, it seems that different levels of strategic, tactical and operational decisions have interaction and cannot be considered independent, multi-level planning is used to integrate strategic and tactical decisions. It should be noted that in order to ensure the effectiveness of the proposed approach, it is compared with the traditional sequential approach used in most articles.

The next section describes the problem that our developed mathematical model seeks to resolve.

## Materials and methods

### Problem description

**Fig 2:**
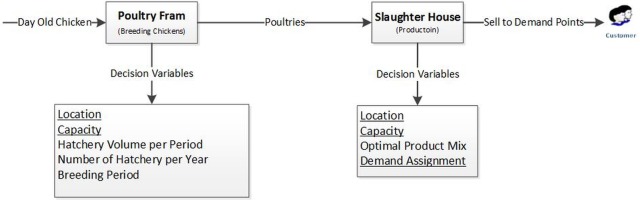
Supply chain structure and decision variables*. * Underlined Variables are Strategic Decisions.

Consider a bi-level supply chain including a poultryfarm and a slaughterhouse. Managing this supply chain includes different decision-making levels such as long-term, medium-term, and short-term. As shown in Fig 2, in this supply chain, inventory flow begins with the purchase of day-old chicks from parent farms. After delivering the day-old chicks, the poultryfarms raises chickens and after the poultries are raised to the desired age, they are sent to the slaughterhouse. At this stage, the slaughterhouse can convert the poultries into different products, which vary in terms of finished expense and sales price; therefore, the slaughterhouse must optimize its product mix per these two factors. It should be noted that locating each of the poultry farms and slaughterhouses and assigning the slaughterhouses to markets are amongst the considerations in the decision-making process However, because this supply chain produces one of the main commodities in the country, determining the price of the final products is the government’s responsibility and cannot be determined by the supply chain.

To facilitate making these decisions simultaneously and to consider the interactions, for example the effect of location on rearing period, this article develops and presents a model based on bi-level programming. According to this model, first at Level 1, long-term decisions are evaluated as leader variables. Then in Level 2, tactical and operational decisions are made.

**Fig 3:**
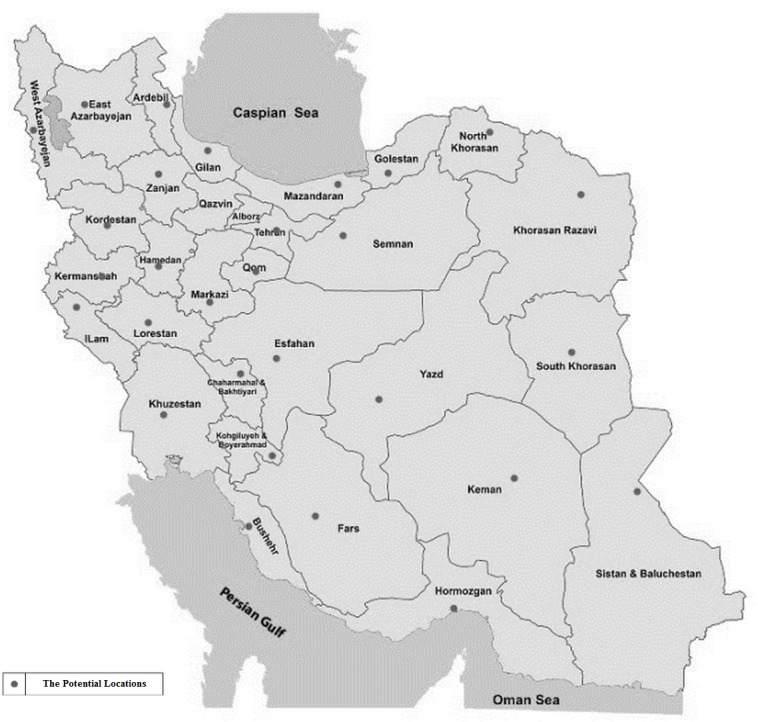
The potential locations for poultryfarms and slaughterhouses.

According to Fig 3, in the first level of decision-making an appropriate place to establish poultryfarms and slaughterhouses should be selected amongst pre-determined locations. Besides, in this phase suitable markets should be determined to sell the products, and the number of products that should be sent to each market should be established. Capacity levels are evaluated in this level as well.

In the second phase of decision-making, tactical and operational decisions are considered. One of the most important decisions made in poultryfarms in this level is the breeding period. By increasing the length of the breeding period, economies of scaled and prorated fixed costs can be used. But by decreasing this age, the total number of breeding periods and, therefore, total annual production can be increased. Also, given that chicken weight growth rate does not increase linearly during the breeding period, the optimal breeding period should be calculated according to this growth curve. Another important consideration is the effect of various locations of poultryfarms on parameters such as growth rate and mortality. Depending on the geographical conditions of different parts of the country, effective parameters in the supply chain such as growth rate, mortality, and expenses may vary. Therefore, decisions should be optimized based on the special conditions of each supply chain.

Other tactical decisions in the poultryfarms include hatching volume in each period, number of breeding periods per year, and the optimal product mix. Hatching volume is the number of day-old chicks that are raised by poultryfarms in each breeding period. Given how old chickens are when slaughtered, the number of breeding periods may vary during the year. However, because all poultry related products are different in terms of finished expense and sales price; in consideration of the market, the optimal combination of the products should be calculated as well. It should be noted that in this article, packaged chicken and cleaned and packaged chicken are considered the producible goods of the slaughterhouse.

The different steps that need to be taken in this supply chain are as follows. First, day-old chickens are bought from the parent poultryfarm and delivered to the broiler poultryfarm location. Then, the breeding stage begins. In this stage, the poultryfarm raises chickens with a proper diet. In addition to feeding cost, this stage involves other costs including vaccines and drugs. After chickens reach the suitable age or weight, they are sent to the slaughterhouse. Between each two breeding periods, about 15 days should be spent on cleaning and preparing the poultryfarm. Then, adult poultries delivered to the slaughterhouse will be slaughtered and converted to the desired products. All stages face some form of deterioration. When the chickens are still alive, there is a mortality rate, and when they are slaughtered they experience weight loss, as blood and other materials exit from the chickens’ bodies. When delivering the product to the demand location, evaporation of water from the chickens’ bodies also causes weight loss. Therefore, special attention to this reduction is essential.

In the next section, a mathematical model based on bi-level programming is proposed to integrate strategic and tactical decisions in poultry supply chain. All parameters and interactions between different types of decisions discussed above, are considered in proposed model.

### Mathematical model

As mentioned, in this paper a mathematical model is presented to integrate the strategic and tactical decisions in poultry supply chain. Bi or multi-level optimization problems are problems that optimize conflicting goals in a hierarchical structure. In such problems, there are multiple decision-making levels, each of which control a part of available decision variables in decision-making [29, 30]. Each level has its own special objective function, and each objective function has its own constraints in each of the hierarchical levels, while there may be shared constraints for the whole problem. Bi-level problems are a special case of multi-level problems and are modeled by considering one or more leaders and one or more followers. Leaders and followers each have their decisions variables and constraints. The main difference between the leader and the follower relates to priority in decision making. The leader determines the feasible space for the follower by defining values of decision variables. However, considering that the leader’s and the follower’s profits are linked, there is a mutual relationship. Thus, problems arise with the use of a unipolar mode and the interests of both levels should be considered. In this article, the concept of bi-level programming is used to integrate strategic, tactical, and operational decisions. Strategic decision variables define the feasible space for tactical and operational variables; therefore, strategic variables are called leaders and tactical and operational variables are considered followers. In other words, in the proposed approach, interactions occur between two levels of decisions, not between two different firms. To follow basic game theory principles, it can be assumed that strategic managers are leaders and operational managers are followers. In the first level, strategic variables like locations should be determined to minimize total cost and market entry index. In the second level, tactical and operational variables should be determined to maximize the profit.

Sets, parameters, and variables of the model are as follows:

Sets:

i: The potential locations for poultryfarms (i = 1,2,…,I)
j: The potential locations for slaughterhouses (j = 1,2,…,J)
l: Demand points (l = 1,2,…,L)
k: Products set (k = 1 for packaged and 2 for packaged and cleaned slaughtered poultry)
c: Poultryfarm capacity set (c = 1,2,…,C)
c’: Slaughterhouse capacity set (c’ = 1,2,…,C’)
*st*: Inventory condition set; st = chn (Day-old Chicken), i.plty (In-way Inventory), plty (Poultry), prdt (Final Product)

Decision variables:

*Hch*.*Vlm*_*i*_: Hatchery volume per period for poultryfarm placed in position i
*N*.*Hch*_*i*_: Number of hatchery per year for poultryfarm placed in position i
*Q*_*jlk*_: Amount of product k sent to demand point l from slaughterhouse j
*T*: Breeding period
*P*_*k*_: Production ratio of product k
${Q_{ij}^{\prime }}_{}$: Amount of mature poultry sent from poultryfarm i to slaughterhouse j (kg)
*x*_*ic*_: 1 if poultryfarm with capacity c locates in i, else 0
$y_{jc^{\prime }}$: 1 if slaughterhouse with capacity c’ locates in j, else 0
*F*_*l*_: 1 if delivered product to demand point l is less than deman, else 0

Parameters:

${{D.Rate}_{i}^{st}}^{}$: Mortality rate of inventory in condition st in location i
*P*^*g*^: Day-old chicken price
*Tr*.*C*^*st*^: Transportation cost per unit for inventory in condition st
*C*_*ic*_; Fixed-cost of poultryfarm location with capacity level c in location i
$C_{jc^{\prime }}$: Fixed-cost of slaughterhouse location with capacity level c’ in location j
*D*_*l*_: Demand level in point l
*CD*: Cost per unit for feed
*V*(*T*): Feed consumption function
*C*.*dly*: Other daily costs of breeding in poultryfarm
*T*.*Co*_*k*_: Ratio of conversion poultry unit to product k
*W*(*T*): Poultry growth function
*P*_*k*_: Unit price of product k
*Dstc*_*gi*_: Distance between parent poultryfarm and meat poultryfarm in point i
*Dstc*_*ij*_: Distance between poultryfarm i and slaughterhouse j
*Dstc*_*jl*_: Distance between slaughterhouse j and demand point l
*AP*^*k*^: Reduction percentage of surplus inventory selling price
*ST*: Time needed for preparation and cleaning poultryfarm between two breeding periods
*PC*_*k*_: Direct cost of production each kilograms of product k
*Cp*_*c*_: Capacity of poultryfarm of type c
${Cp}_{c^{\prime }}$: Capacity of slaughterhouse of type c’
*Wl*_*k*_: Percentage of poultry weight loss in slaughtering
*TWl*_*k*_: Percentage of weight loss in transporting products

Model assumptions:

- The number of chickens raised in different breeding periods is equal.
- A breeder poultryfarm exists in each province.
- Poultry and slaughterhouse that are established in one province will be located in a predetermined distance.
- Selling products in markets that have more demands is easier.
- The desired time span is limited and equals to one year.
- In case of producing more than the market need, extra products will be sold with APk percentage of the original price.
- Various weather conditions do not impact the growth and feed consumption rate.

Given these parameters and variables, the mathematical model of the problem will be as follows:

*[1st level]*

$$\begin{array}{l} Min\left[\sum_{i}\sum_{c}c_{ic}x_{ic}+\sum_{j}\sum_{c^{\prime }}c_{jc^{\prime }}y_{jc^{\prime }}\right]+\sum_{i}^{}\sum_{c}^{}\left[\left(N.Hch_{i}\;*\;Hch.Vlm_{i}\right)\left(1+D.Rate_{i}^{chn}\right)Dstc_{gi}Tr.C^{chn}\right]\\ +\left[\left(N.Hch_{i}\;*\;Hch.Vlm_{i}\right)\;*\;D.Rate_{i}^{plty}\;*\;Tr.C^{plty}\right]+\sum_{k}^{}\sum_{l}^{}\sum_{j}^{}Q_{jlk}\;*\;Dstc_{jl}\;*\;Tr.C^{pdct} \end{array}$$ (1)

$$Min\frac{\sum_{l}^{}\sum_{k}^{}\sum_{j}^{}Q_{jlk}Dstc_{jl}}{D_{l}}$$ (2)

*[2*^*nd*^ *level]*

$$\begin{array}{l} Max\left\{\left[\sum_{k}\sum_{l}\sum_{j}\left(Q_{jlk}\;*\;(1-WL_{k})-D_{l}\right)\right]\left[\left(1-AP_{k}\right)P_{k}\right]+\left[\sum_{k}\sum_{l}D_{l}\;*\;P_{k}\right]\right\}\left(1-F_{l}\right)+\\ \left[\sum_{k}\sum_{l}\sum_{j}Q_{jlk}\;*\;(1-WL_{k})P_{k}\right]F_{l}+\left(N.Hch_{i}-1\right)ST-\left(Hch.Vlm_{i}\;*\;N.Hch_{i}\left(1+D.rate_{i}^{chn}\right)p^{g}\right)-\\ \left(PC_{k}\;*\;\sum_{l}\sum_{k}\sum_{j}Q_{jlk}\right)-\left(Hch.Vlm_{i}\;*\;N.Hch_{i}\int_{0}^{T}CD\;*\;V\left(T\right)dt\right)-\left(Hch.Vlm_{i}\;*\;N.Hch_{i}\;*\;C.dly\;*\;T_{i}\right) \end{array}$$ (3)

Subject to:

$$\left[\sum_{k}\sum_{j}^{}Q_{jlk}-D_{l}\right]+MF_{l}\geq 0\;,\forall l$$ (4)

$$\left[-\sum_{k}\sum_{j}^{}Q_{jlk}+D_{l}\right]+M(1-F_{l})\geq 0\;,\forall l$$ (5)

$$\sum_{k}\sum_{l}\sum_{j}^{}Q_{jlk}\left(1+T.Co_{k}\right)\leq Q_{ij}^{'}\;,\forall j$$ (6)

$$Q_{ij}^{'}\leq \left(Hch.Vlm_{i}\;*\;N.Hch_{i}\right)W\left(T\right)(1-D.rate_{i}^{i.plty})\left[\frac{i}{j}\right]\left[\frac{j}{i}\right]\;,\forall i,j$$ (7)

$$\sum_{k}^{}\sum_{l}^{}Q_{jlk}\leq My_{jc^{\prime }}\;,\forall j$$ (8)

$$Hch.Vlm_{i}\leq \sum_{i}^{}\sum_{n}^{}Cp_{n}x_{in}\;,\forall i$$ (9)

$$Hch.Vlm_{i}\left(D.Rate_{i}^{plty}\right)(D.rate_{i}^{i.plty})\leq \sum_{j}^{}\sum_{c^{\prime }}^{}Cp_{c^{\prime }}y_{jc^{\prime }}$$ (10)

$$\left(N.Hch_{i}\;*\;T_{i}\right)+\left(N.Hch_{i}-1\right)ST\leq 365$$ (11)

$$\sum_{c}^{}x_{ic}=\sum_{c^{\prime }}^{}y_{jc^{\prime }}\;\forall i=j$$ (12)

$$\sum_{i}\sum_{c}c_{ic}x_{ic}+\sum_{j}\sum_{c^{\prime }}c_{jc^{\prime }}y_{jc^{\prime }}\leq TF$$ (13)

$$Hch.Vlm_{i},N.Hch_{i},Q_{jlk},T_{i},P_{jk},Q_{ij}^{'}\geq 0\;\forall i,n,j,l,k$$ (14)

$$x_{in},x_{jn},F_{l}\in \left\{0,1\right\}\;\forall i,n,j,l,k$$ (15)

**Fig 4:**
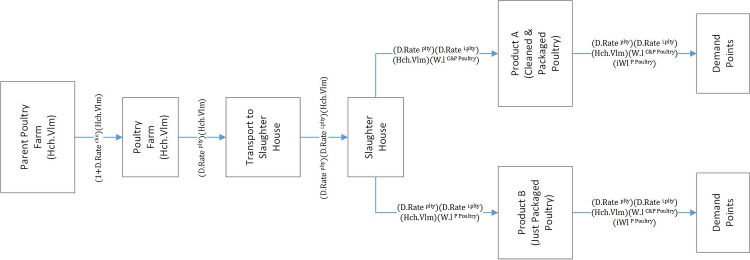
Product flow volume.

The first level involves strategic decision variables while the second level includes tactical and operational variables. The upper level has two objective functions. The first objective function is to minimize the cost related to making strategic decisions. The first and the second terms of the first objective function represent the fixed cost of locating meat poultryfarms and slaughterhouses. The third term represents total annual costs of transportation for day-old chicks from parent poultryfarms to meat poultryfarms. Considering the possibility of mortality while transporting the day-old chicks, meat poultryfarms must purchase more chicks than needed; thus, coefficient (1+D.Ratechn) is multiplied in this term. Fig 4 illustrates the product volume flow in different stages. This volume is determined according to the desired hatching volume of the poultry in poultryfarms.

The fourth term of objective function provides the cost of transporting raised chickens from meat poultryfarms to the slaughterhouses. Because D.Rate^plty^ percent of chickens would die during the breeding period, to calculate the number of raised mature chickens at the end of the period, mortality rate should be multiplied with the number of raised chickens. The fifth term calculates the cost of transporting final products from the slaughterhouses to the demand points. The second objective function of the first level deals with the cost of entering in new markets. The slaughterhouses can choose different markets to provide their products, but the costs of entering these markets and selling the products are different. According to model assumptions, the more potential demand in a market, the less the cost of entering that market. Considering demand volumes and the distance between slaughterhouses and demand points, the objective functions’ structure tries to send the products to nearer markets with more unmet demands.

The model’s second level concerns tactical and operational variables. In this level, the main objective is maximizing the annual profit. First and second terms demonstrate total income of the supply chain. The first term is a case when the volume of the sent product is more than the market demand, and the second term is a case where the sent product is less than the market demand. Because slaughtered chickens lose weight during transportation for different reasons such as evaporation of water in the body, there is a weight difference between the sent products from the slaughterhouse and the amount of delivered products in the market. The impact of this weight loss is applied by multiplying (1-WL_k_) in the amount of sent products. On the other hand, in conditions where the amount which is sent to the market is more than market demands, surplus products are sold with *AP*_*k*_ percent reduction of their real price. The third term demonstrates the income in conditions where the amount of the sent products is most equal to demands. The fourth term demonstrates total cost of preparation and cleaning the poultry between the two hatching periods. Obviously, the number of cleaning periods is one unit less than the number of hatching periods.

The fifth term demonstrates the total cost of buying day-old chickens in a year. Because day-old chickens face mortality during transportation, the number of chickens bought must be (1 + *D*.*rate*^*chn*^) percent more than the desired number of chicks for raising. The sixth term illustrates the total direct cost of production for each kilogram of product. The seventh term is the total cost of feed consumption in the breeding period. To measure the feed consumption of each chicken, an integral of chicken feed consumption function overtime should be used. Finally, the eighth term demonstrates other daily production costs in the poultryfarms such as vaccine costs. Direct costs of poultry farming, which include buying day-old chickens and feeding are considered as well.

**Fig 5:**
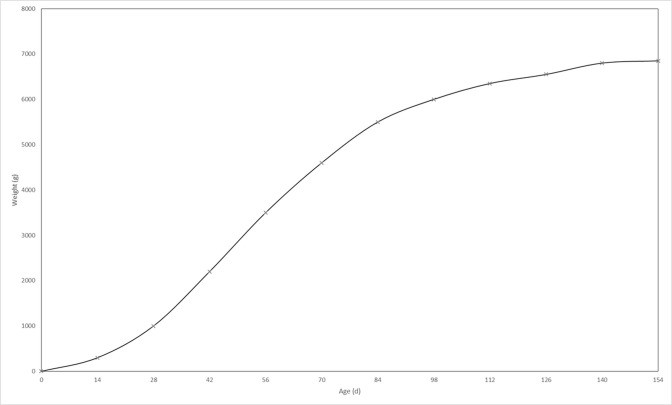
Poultry growth curve [31].

Constraints 4 and 5 determine the correct amount for the Tlk binary variable. In case the total supply of each product is more than its total demand, the variable will be zero; otherwise, it will be one. Constraint 6 guarantees the balance between sending and receiving products in slaughterhouses. Since conversion takes place in the slaughterhouse to produce each product unit (kg.), more than one chicken unit (kg.) is needed; therefore, the conversion ratio of any of these products should be considered. Constraint 7 guarantees this balance in the poultryfarm and illustrates that total sending from the poultryfarm should not be more than its total production. As shown in the mathematical model, total production of the poultryfarm equals the number of hatching periods multiplied by hatching volume. In this case, the mortality rate of the chickens should be considered as well because the volume considered for production will be different based on chickens’ final volume, and the number decreases due to mortality. In addition, since the amount of sent product from poultry to the slaughterhouse is calculated by weight, the right side of the equation must also be expressed in terms of weight; therefore, total weight of the poultry’s production each year equals the number of raised chicks (the number of hatching periods multiplied by hatching volume in each period) multiplied by the rate of chicks that stay alive and multiplied by an integral of weight function over time. The chicken weight over time can be expressed by a weight function. Fig 5 shows the poultry growth curve:

It should be noted that term $\left[\frac{i}{j}\right]\left[\frac{j}{i}\right]$ guarantees that $Q_{ij}^{'}$ would be positive, only if i = j. In other words, slaughterhouses can receive products only from poultryfarms in same province. This is because of governmental laws.

Constraint 8 guarantees that products must only be sent from where the slaughterhouse is established. Considering there are different facilities with different capacities, constraint 9 ensures that the production amount of the poultryfarm should not be more than its capacity. Inequality 10 also demonstrates the capacity for slaughterhouse units. According to this constraint, total products received from poultryfarms for each slaughterhouse should be at most equal to the capacity of each slaughterhouse. Regarding the mortality rate while breeding the chickens and transporting the poultries to the slaughterhouses, the impact of mortality rate should be applied. Constraint 11 demonstrates total time spent for hatching during the year, which is equal to multiplying the number of hatching periods in the breeding period plus the time needed for preparation of the poultryfarm between hatching periods, which should be less than 365 days. Since Iran does not allow the movement of live poultry among different provinces, Constraint 12 guarantees that poultry and the slaughterhouse are established in the same province so there is no need for moving the live poultry among provinces. Constraint 13 demonstrates the total fund limit. Constraints 14 and 15 also show the variables’ value interval.

### Solution method

Perhaps one of the most usable methods to solve multi-objective, multi-level problems, is the fuzzy mathematical planning technique [4, 5]. One of the most popular approaches among fuzzy mathematical planning methods is fuzzy goal programming. In this article, the 11-step fuzzy goal programming algorithm developed by Baky is used to solve the developed mathematical model [13].

The steps of this algorithm are as follows:

Step 1) Solving objective functions and their constraints independently to calculate maximum and minimum values
Step 2) Use upper tolerance limits of goals for each level
Step 3) Calculate the weights:

wij+=1(uij−gij),i=0,1,…,p,j=1,2,…,mi (16)

Step 4) Set l = 1
Step 5) Evaluate membership functions
Step 6) Develop the below model for each for levels and solve it

$$\min\;z=\sum_{j=1}^{m_{0}}w_{0j}^{+}d_{0j}^{+}$$ (17)

$$subject\;to\;\frac{u_{0j}-(c_{1}^{0j}x_{1}+c_{2}^{0j}x_{2}+\cdots +c_{p}^{0j}x_{p})}{u_{0j}-g_{0j}}+d_{0j}^{-}-d_{0j}^{+}=1,\;j=1,2,\ldots ,m_{0}$$ (18)

$$A_{1}x_{1}+A_{2}x_{2}+\cdots +A_{p}x_{p}\left(\begin{array}{c} \leq \\ =\\ \geq \end{array}\right)b,\;x\geq 0$$ (19)

$$d_{0j}^{-}\times d_{0j}^{+}=0\;and\;d_{0j}^{-},d_{0j}^{+}\geq 0,\;j=1,2,\ldots ,m_{0}$$ (20)

Step 7) Elicit variable vector uniting maximum and minimum tolerances
Step 8) Calculate the weights and membership functions:

wKL=1tKLandwKR=1tKR,k=1,2,…,n0 (21)

Step 9) if l< = p-1 go to step 5; otherwise go to step 10
Step 10) Evaluate the weights and members’ functions of objective functions in p-th level
Step 11) Develop below model for problem and solve it

$$\frac{x_{0k}-(x_{0k}^{*}-t_{k}^{L})}{t_{k}^{L}}+d_{0K}^{L-}-d_{0K}^{L+}=1,\;k=1,2,\ldots ,n_{0}$$ (22)

$$A_{1}x_{1}+A_{2}x_{2}+\cdots +A_{p}x_{p}\left(\begin{array}{c} \leq \\ =\\ \geq \end{array}\right)b,\;x\geq 0$$ (23)

$$d_{ij}^{-},d_{ij}^{+}\geq 0\;with\;d_{ij}^{-}\times d_{ij}^{+}=0,\;i=0,1,\ldots ,p,\;j=1,2,\ldots ,m_{i}$$ (24)

$$d_{0K}^{L-},d_{0K}^{L+}\geq 0\;with\;d_{0K}^{L-}\times d_{0K}^{L+}=0,\;k=1,2,\ldots ,n_{0}$$ (25)

$$d_{0K}^{R-},d_{0K}^{R+}\geq 0\;with\;d_{0K}^{R-}\times d_{0K}^{R+}=0,\;k=1,2,\ldots ,n_{0}$$ (26)

To solve the mathematical model, it is first linearized according to S1 Appendix, then converted to FGP using [13] and then solved with GAMS. Real data received from the Statistical Center of Iran is used to solve the model. Computational results are described in Section 5.

## Discussion

**Fig 6:**
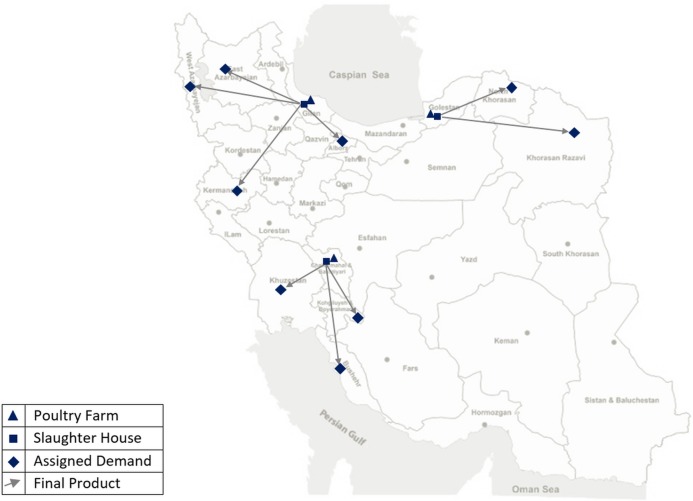
Supply chain’s structure using Bi-level programming.

**Fig 7:**
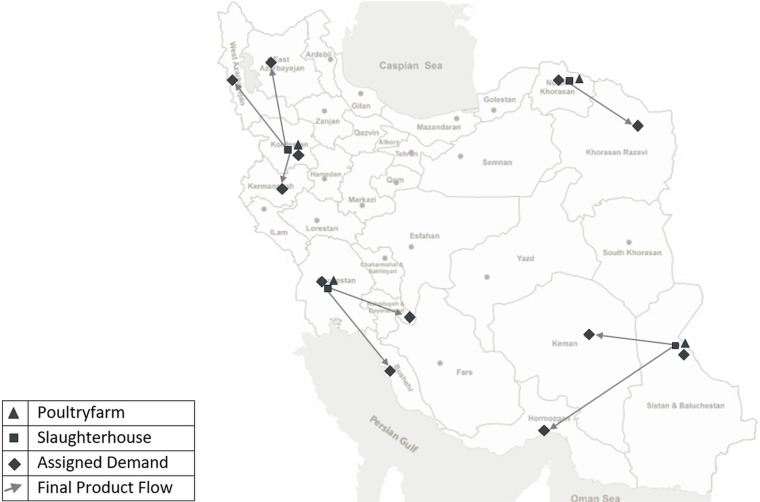
Supply chain's structure under sequential DM.

After linearizing model, to evaluate the proposed approach in this article, the problem is modelled and solved using multi-level programming and is then modelled and solved using a traditional sequential approach. In the sequential approach, decisions of different levels are made independently and results of upper decisions enter the next level as a parameter. In other words, in the sequential approach, the first decision about the supply chain’s structure is made, which includes the location and capacity of the poultryfarms and the slaughterhouses and the type of market allocation are determined. In the next stage, tactical and operational variables such as breeding period are evaluated. Fig 6 and Fig 7 represent the supply chain’s structure in bi-level and sequential approaches, respectively.

As shown in Fig 6 and Fig 7, although the total fund is equal in the two approaches, bi-level programming results in three poultryfarms and three slaughterhouses while sequential programming results in four poultryfarms and four slaughterhouses. The locations selected in the bi-level approach are more expensive and more appropriate for breeding. According to historical data, death rate in these locations are less than in locations selected using the sequential approach. Locations with lower fixed investment costs, and thus, higher death rates, are selected in the sequential approach. This is the result of considering interactions among different decision types.

Table 2 compares cost indicators in the bi-level and sequential approaches:

**Table 2:** Costs and income in bi-level and sequential DM process.

	Fixed Cost	Annual variable Cost	Annual Income
**Bi-level Programming**	26,622,000,000.00	8,706,310,768.19	15,883,114,773.35
**Independent Decision Making**	26,372,040,000.00	9,463,273,238.56	15,545,661,722.14

**Fig 8:**
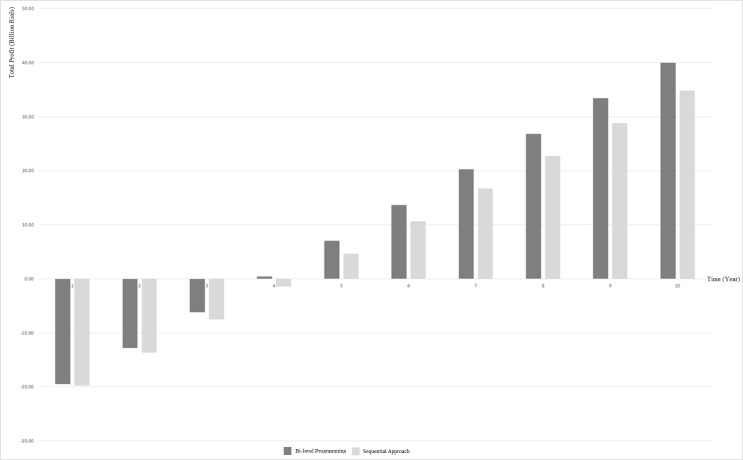
Commulative profit/time in bi-level and sequential decision-making.

As shown in Table 2, the total fixed cost in the sequential case is slightly less than the bi-level case, while annual variable cost and income in the bi-level case is better. Although the time span considered for solving mathematical model is equal to one year, in order to emphasis on the differences between the results of sequential approach and bi-level approach, 10-year analysis is presented too. Fig 8 demonstrates the commulative profit in the bi-level and sequential approaches in ten years. According to Fig 8, the difference in commulative profit grows over time. Insofar as in the 10th year commulative profit using bi-level programming is about 5 billion rials more than the sequential approach. This illustrates the efficiency of bi-level programming over time.

One of the main reasons for more annual profit using the bi-level decision-making approach is the consideration of tactical and operational variables while deciding strategic variables. For example, consider the breeding period. The average optimal breeding period using bi-level and sequential approaches respectively, would be 43.5 and 46 days. Differences in optimal breeding periods between the two approaches stem from the selected locations. For example, one of the selected locations in the bi-level approach is Gilan. Gilan has a humid climate, which is suitable for raising chickens. The mortality rate in this province is about 10 percent, while this rate is about 20 percent in Khuzistan, which is one of the selected locations in the sequential approach. Therefore, to avoid incurring the cost of upper mortality rate, the breeding period would be longer, and the number of chickens raised annually will reduce. In other words, the less poultry raised annually, the lower annual poultry losses.

Table 3 compares optimal variables’ values in the bi-level and sequential approaches:

**Table 3:** Optimal values for decision variables in bi-level and sequential approach.

	Poultry Farm & Slaughter House Location	Capacity (Unit)	Products Ratio		Assigned Demand Point	Delivered Product Ratio	No. of Hatchery per Year	Breeding Period
			packaged	Packaged and Cleaned				
**Bi-level DM**	Golestan	100.000	0	1	Khorasan Shomali	0.22	6.29	43
					Khorasan Razavi	0.78		
	Gilan	80.000	0	1	Azarbayejan Sharghi	0.32	6.29	43
					Azarbayejan Gharbi	0.24		
					Kermanshah	0.28		
					Alborz	0.17		
	Chaharmahale Bakhtiari	100.000	0	1	Kohkilooye va Bovirahmad	0.17	6.19	44
					Khuzestan	0.62		
					Bushehr	0.21		
**Sequential Approach**	Systano Baluchestan	100.000	0	1	Hormozgan	0.22	5.79	48
					Kerman	0.32		
					Systano Baluchestan	0.46		
	Khuzestan	100.000	0	1	Kohkilooye va Bovirahmad	0.17	5.89	47
					Bushehr	0.62		
					Khuzestan	0.21		
	Kordestan	100.000	0	1	Kermanshah	0.32	6.19	44
					Azarbayejan Gharbi	0.24		
					Azarbayejan Sharghi	0.28		
					Kermanshah	0.17		
	Khorasane Shomali	80.000	0	1	Khorasane Shomali	0.24	6.08	45
					Khorasane Razavi	0.76		

As shown in this table, selected locations for poultryfarms and slaughterhouses are different in bi-level and sequential approaches. One of the reasons for this difference is that the sequential approach solves the problem by minimizing location and allocation costs, while the bi-level approach considers the objective of the model’s second level, which are tactical and operational variables such as optimal breeding period. Another important point is the difference in breeding period between the two approaches.

As mentioned, selected provinces for establishing poultryfarms and slaughterhouses in the bi-level and sequential decision-making processes have very different climates. A province like Gilan is mild and humid while Khuzestan is warm and dry. Therefore, in similar conditions, the mortality rate will be much higher in Khuzestan. According to computational results, the higher the mortality rate, the longer the optimal breeding period, and therefore, the lower the number of hatchery per year. Another important point is the optimal ratio of each product. Obviously, according to the outputs in both approaches, all slaughtered poultries should be converted to “packaged and cleaned chicken” production and then sent to demand points not in “just packaged chicken”. The selling prices of just packaged and packaged and cleaned chickens are more than cleaning process costs, so it is economical for the supply chain to sell a packaged and cleaned product. Moreover, the differences between breeding periods in different provinces should be noted. Due to the costs of poultryfarms and slaughterhouses, the day-cost of keeping a chicken is about 566 rials, so it is important to optimize the breeding period because keeping chickens for one extra day in the supply chain can incur a cost of up to 215,210,000 rials. As shown in Table 2, the most important difference between bi-level and sequential approaches is their costs. Even though the difference in income in the two approaches appears inconsequential, a considerable difference in annual cost renders the sequential approach to yield much less in total profit than the bi-level approach in the long term.

**Fig 9:**
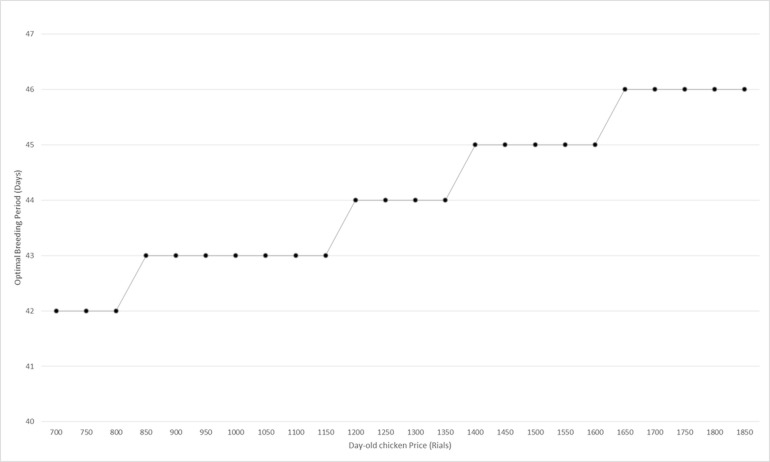
Optimal breeding period in day-old chicken price fluctuation.

One of the most important problems that poultry supply chains face is price fluctuation. This fluctuation occurs across components of the supply chain. Feed and day-old year prices are two important factors with the most price fluctuation. So, the effect of these factors’ fluctuations on different decision-making variables and objective functions should be considered. Historical data shows that day-old chicken price fluctuations in the past five years vary between 7000 to 18500 rials. Fig 9 shows the sensitivity analysis on this factor.

As illustrated by this Fig 9, day-old chickens’ price fluctuation affects the optimal breeding period. According to this Fig 9, the function of optimal breeding period on day-old chicken price is increasing. It should be noted that this function is increasing, not strictly increasing. Increasing day-old chickens’ price when the final product price is fixed encourages poultryfarms to decrease breeding periods per year to purchase less total chickens per year, so the breeding period increases.

**Fig 10:**
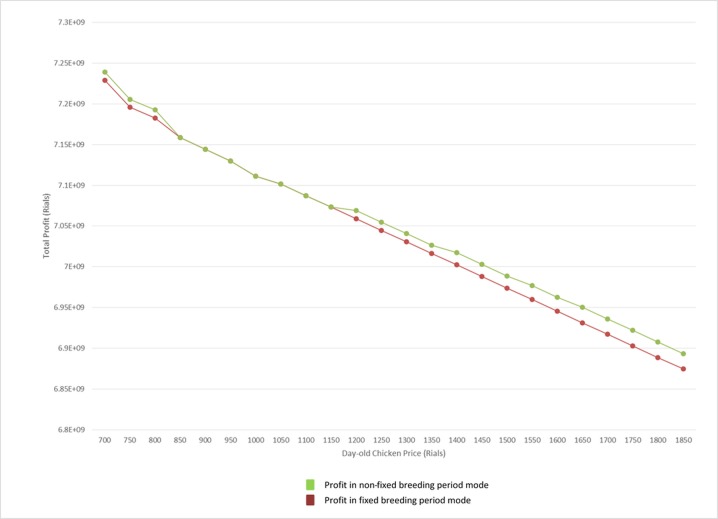
Profit comparison in fixed and non-fixed breeding period.

Unlike this analysis, in the real-world, the breeding period in supply chains is considered fixed. Fig 10 shows the difference between total costs in the presence of day-old chicken price fluctuation in two cases: fixed breeding period and when breeding period is determined according to day-old chicken price.

As is shown in Fig 10, the total cost in variable breeding period case is always less than the case of a fixed breeding period except for in a period in which the optimum breeding period is equal in two cases.

**Fig 11:**
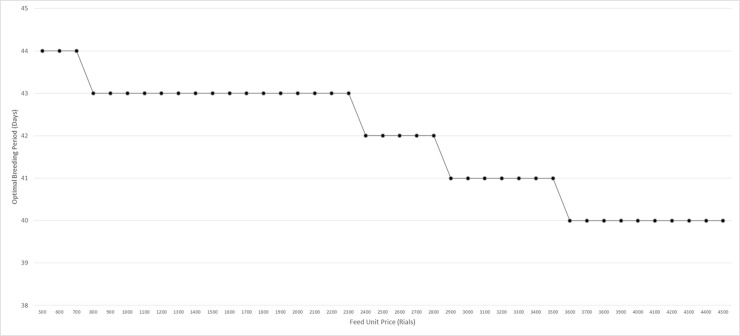
Optimal breeding period in feed price fluctuation.

Another important factor is feed price. Fig 11 shows that feed prices’ fluctuation affect the optimum breeding period.

**Fig 12:**
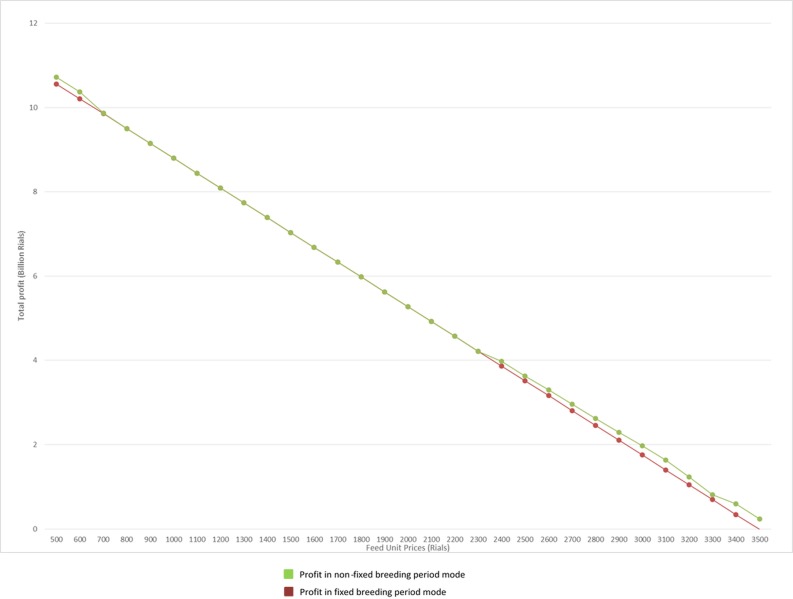
Profit comparison in fixed and non-fixed breeding period (feed price fluctuation).

As is shown, increasing the feed price leads to shorter breeding periods. Older poultries result in more feeding volume, and thus incur more feeding costs per day. Per the growth rate curves in Fig 5, after 42 days, as the poultry age increases, the weight gain rate decreases. On the other hand, feeding volume per day will increase exponentially as the age increases. So, poultry farms tend to decrease the feeding period. Fig 12 compares two policies facing with regards to feed fluctuation. As is shown in Fig 12, changing the breeding period results in more annual profit. It is assumed that the price of the final product is fixed.

## Conclusion

In this paper, livestock inventory is presented as a type of inventory that has major differences with other types of inventory such as perishable products. Despite the importance of livestock inventory in human life, research studies often neglect it. When livestock inventory is researched, different levels of decisions are made sequentially. Thus, in this paper, a bi-level programming model is presented to optimize the variables in strategic, tactical, and operational levels in Iran’s meat poultry supply chain as one of the most important livestock supply chains in the country. The computational results indicate that the bi-level approach works more efficiently than the sequential approach. Other managerial insights from the computational results are listed below:

- Considering the interactions among different decision levels in the supply chain results in more profit and lower cost.
- Despite the dominant approach, livestock specific decision variables like breeding period are not predetermined and fixed. Rather, these decisions should be made according to environmental conditions like poultryfarm location.

Livestock supply chains should be designed as agile to react to changes in parameters like feeding price, because these shifts can result in less costs.

## Supporting information

S1 Dataset Related dataset. (XLSX)

S1 Appendix Linearizing model. (DOCX)
