# Supplementary material for: Integrating strategic and tactical decisions in livestock supply chain using bi-level programming, case study: Iran poultry supply chain
Source: PLoS One. 2017 Oct 5;12(10):e0185743. doi: 10.1371/journal.pone.0185743 (PMC5628908; doi:10.1371/journal.pone.0185743)
Supplement: S1 Appendix — (DOCX) [file pone.0185743.s002.docx]

**S1- Appendix: Linearizing Model**

To linearize the proposed model, first the auxiliary variables with the following characteristics must be defined:

$$W=N.Hch*Hch.Vlm$$

$$Z_{ic}=W*x_{ic}$$

$$R_{jlk}=Q_{jlk}*T_{l}$$

$$O_{\tau}=W*D_{\tau}$$

$$h_{\tau}=N.hch*D_{\tau}$$

$$D_{\tau} \left\{ \begin{aligned} 1 if feeding period is equal to \tau\\ 0 Else \end{aligned} \right\}$$

Regarding the information of the case study, the decision variables’ limits are considered as follows:

$$N.Hch\leq10$$

$$Hch.Vlm\leq35000$$

$$Q_{jlk}\leq2240000$$

One of the phrases in the model is$\int_{0}^{T} CG*G\left( T \right)dt$. In real situations, the breeding period is always considered an integer number. To linearize the mentioned term, the weight of poultry on each day of the breeding period is considered as a tabular function and is multiplied in covariable D_τ_. This variable indicates the length of the breeding period. When the length of the period equals τ, this variable will be one; otherwise, it will be zero.

Using the limits and covariables and according to [[32](#_ENREF_32)] , the linearized model is as follows:

| 1^st^ Level |  |
| --- | --- |
| $Min \left[\sum_{i} \sum_{c} c_{ic}x_{ic}+\sum_{j} \sum_{c'} c_{jc'}y_{jc'}\right]+\sum_{i} \sum_{c} \left[\left(1+D.Rate^{chn}\right)Dstc_{g_{i}i}Tr.C^{chn}\right]Z_{ic}$ $+\left[W*D.Rate^{plty}*Tr.C^{plty}\right]+\sum_{k} \sum_{l} \sum_{j} Q_{jlk}*Dstc_{jl}*Tr.C^{pdct}$   | *(1)* |
| *$Min \frac{\sum_{l} \sum_{k} \sum_{j} Q_{jlk}Dstc_{jl}}{D_{l}}$* | *(2)* |

| 2^nd^ level |  |
| --- | --- |
| $Max\left\{\left\{\left[\sum_{k} \sum_{l} \sum_{j} \left(Q_{jlk}*(1-WL_{k})-D_{l}\right)\right]\left[\begin{matrix} \\ \left(1-AP_{k}\right)P_{k} \\ \end{matrix}\right]+\left[\sum_{k} \sum_{l} D_{l}*P_{k}\right]\right\}\right.$ $-\left\{\left[\sum_{k} \sum_{l} \sum_{j} \left(R_{jlk}(1-WL_{k})-T_{l}.D_{l}\right)\right]\left[\begin{matrix} \\ \left(1-AP_{k}\right)P_{k} \\ \end{matrix}\right]+\left[\sum_{k} \sum_{l} D_{l}*P_{k}\right]T_{l}\right\}$ $\left.+\left[\sum_{k} \sum_{l} \sum_{j} R_{jlk}*(1-WL_{jk})P_{k}\right]\right\}$ $+\left(N.Hch_{ }-1\right)ST$ $-\left\{\left(W\left(1+D.rate^{chn}\right)p^{chn}\right)\right.$ $\left.-\left(PC_{k}*\sum_{l} \sum_{k} \sum_{j} Q_{jlk}\right)+\left(\sum_{\tau=1}^{75} CG_{\tau}.O_{\tau}\right)+\left(\sum_{\tau=1}^{75} C.dly.\tau.D_{\tau}\right)\right\}$       | *(3)* |

| Subject to |  |
| --- | --- |
| *$\left[\sum_{k} \sum_{j} Q_{jlk}-D_{l}\right]+MT_{l}\geq0 ,\forall l$* | *(4)* |
| *$\left[-\sum_{k} \sum_{j} Q_{jlk}+D_{l}\right]+M(1-T_{l})\geq0$* | *(5)* |
| *$\sum_{k} \sum_{l} \sum_{j} Q_{jlk}\left(1+T.Co_{k}\right)\leq Q' ,\forall j$* | *(6)* |
| $Q'\leq O_{\tau}.WT_{\tau}(1-D.rate^{i.plty}) ,\forall i$ | *(7)* |
| $\sum_{k} \sum_{l} Q_{jlk}\leq My_{jc'} ,\forall j$ | *(8)* |
| *$Hch.Vlm\leq\sum_{i} \sum_{n} Cp_{n}x_{in} ,\forall i$* | *(9)* |
| *$Hch.Vlm\left(D.Rate^{plty}\right)(D.rate^{i.plty})\leq\sum_{j} \sum_{c'} Cp_{c'}y_{jc'}$* | *(10)* |
| *$\left(\tau O_{\tau}\right)+\left(N.Hch_{i}-1\right)ST\leq365$* | *(11)* |
| *$\sum_{c} x_{ic}=\sum_{c'} y_{jc'} \forall i,j$* | *(12)* |
| *$\sum_{n} \sum_{i} x_{ic}=1$* | *(13)* |
| *$\sum_{m} \sum_{j} y_{jc'}=1$* | *(14)* |
| *$W\leq10Hch.Vlm$* | *(15)* |
| *$W\leq35000N.Hch$* | *(16)* |
| $W\geq10Hch.Vlm+35000N.Hch-350000$ | *(17)* |
| $Z_{ic}\leq35000x_{ic}$ | *(18)* |
| $Z_{ic}\leq W$ | *(19)* |
| $Z_{ic}\geq W-\left(1-x_{ic}\right)350000$ | *(20)* |
| $R_{jlk}\leq2240000T_{l}$ | *(21)* |
| $R_{jlk}\leq Q_{jlk}$ | *(22)* |
| $R_{jlk}\geq Q_{jlk}-2240000\left(1-T_{l}\right)$ | *(23)* |
| $O_{\tau}\leq350000D_{\tau}$ | *(24)* |
| $O_{\tau}\leq W$ | *(25)* |
| $O_{\tau}\geq W-350000\left(1-D_{\tau}\right)$ | *(26)* |
| $h_{\tau}\leq10D_{\tau}$ | *(27)* |
| $h_{\tau}\leq N.hch$ | *(28)* |
| $h_{\tau}\geq N.Hch-10\left(1-D_{\tau}\right)$ | *(29)* |
| $Hch.Vlm, N.Hch, Q_{jlk}, T, P_{jk}, Q', W, Z_{ic}, R_{jlk}, O_{\tau}, h_{\tau}\geq0 \forall i,n,j,l,k$ | *(30)* |
| $x_{ic}, y_{jc'}, T_{l}, D_{\tau} \in\left\{0,1\right\} \forall i,c, c', j,l$ | *(31)* |
